# Effect of nitrogen and phosphorus application on starch characteristics and quality of rice with different nitrogen efficiency

**DOI:** 10.3389/fnut.2024.1462689

**Published:** 2024-10-22

**Authors:** Guotao Yang, Qin Wang, Guohao Zhang, Chunyan Jiang, Peng Ma, Yungao Hu

**Affiliations:** College of Life Science and Engineering, Southwest University of Science and Technology, Mianyang, China

**Keywords:** rice quality, physicochemical properties of starch, starch structure, nitrogen and phosphorus application, different nitrogen-efficient rice varieties

## Abstract

**Introduction:**

The application of nitrogen and phosphorus fertilizers is an important factors affecting the quality of rice, and different nitrogen-efficient rice varieties show significant differences in their response to nitrogen and phosphorus application.

**Methods:**

In this experiment, a low-nitrogen-high efficiency variety (Deyou 4727) and a high-nitrogen-high efficiency variety (Jingyou 781) were selected, and the changes in rice quality and differences in starch structure under nitrogen–phosphorus interaction treatments were determined.

**Results:**

Appearance, flavor, starch content and thermodynamic properties, endosperm cell arrangement, and starch granule morphology and size were significantly influenced by variety, nitrogen-phosphorus interactions, and their interaction effects. The effect of nitrogen fertilizer on quality was greater than that of phosphorus fertilizer. The whiteness and chalkiness rates of Deyou 4727 first decreased and then increased with increasing nitrogen fertilizer application, wheras the appearance quality of Jingyou 781 increased with increasing nitrogen fertilizer application. Starch crystallinity in Deyou 4727 first increased and then decreased with increasing nitrogen fertilizer application, whereas starch crystallinity in Jingyou 781 increased continuously with increasing nitrogen fertilizer application. The application of phosphorus fertilizer led to an increase in starch crystallinity in both nitrogen-efficient rice varieties, consistent with the response of different rice varieties to nitrogen and phosphorus in terms of appearance and chalkiness. With the increasing application of nitrogen and phosphorus fertilizers, the differences in the physicochemical properties and structure of starch became more significant.

**Discussion:**

High-nitrogen-efficient rice varieties can significantly improve appearance quality under high nitrogen conditions, and the interactions of medium-high nitrogen and low-medium phosphorus can lead to a significant decrease in starch thermal parameters and retention rate, thus improving rice cooking quality. Low-nitrogen-efficient rice varieties can also improve the quality of rice under low-medium-nitrogen conditions with appropriate application of phosphorus fertilizer.

## 1 Introduction

Rice, one of the world’s major food crops, is consumed as a staple food by more than half of the population of China (1). With the development of hybridization technology, the total yield of rice in China has significantly increased; however, improvement in rice quality lags behind the increase in yield. Currently, with the increase in the living standard in China and, consequently, increased food consumption, the demand for higher quality rice has increased; thus, quality has become as important a factor as yield in rice production (2, 3). The quality of rice is a combination of characteristics: appearance, cooking process, nutrition, and physicochemical properties. Among these four characteristics, appearance and physicochemical properties are the most important in terms of improving quality and are reflected in the quality of the cooked product. The palatability of the rice can be quantified and assessed by measuring certain properties: starch content, the internal structure of the rice grain, and physicochemical properties, all of which rice plays important roles in its nutritional and cooking quality (4). The amylose content in rice is mainly influenced by genetic characteristics, which are controlled by *Wx* genes and are reflected in the food quality of rice (5). However, the amylose content (and thus the food quality) of rice is determined by genes and environmental conditions—such as temperature, light, water, and air—during the growth period (6–9). Under the same external conditions, such as the local climate and cultivation system, field water and fertilizer regulation technology are the main factors affecting rice quality (10–12).

Nitrogen, the major mineral element required for plant growth, is the most common limiting factor for individual plant growth in either natural or cultivated ecosystems. Phosphorus is essential for normal plant growth (13). The addition of phosphorus, a component of nucleic acids, promotes cell division and proliferation, thus significantly increasing the growth of rice roots (14). Different types of fertilizers, such as nitrogen and phosphorus, can significantly affect rice quality. Many studies have been conducted on the effects of nitrogen fertilizer applications on rice quality (15, 16). Wang et al. (17) reported that the nitrogen application rate had a negative effect on the appearance quality of rice: with an increase in the nitrogen application rate, the degree of chalkiness of the grain increased significantly. Research by Song et al. (18) showed that as the nitrogen application rate increased, the viscosity of rice gel decreased, protein content increased, and the amylose content of the grains decreased. However, it has been reported in a few studies that nitrogen application can significantly increase the amylose content of rice (19). Therefore, adjustments to the quantity of nitrogen applied to the rice crop can improve the nitrogen absorption and utilization efficiency of rice, reduce environmental pollution, and have a synchronous effect on improving rice yield and quality (20). Currently, there is relatively little research on the impact of phosphorus application on rice quality. Increasing the phosphorus application can improve brown rice and whole rice rates, and reduce the peak viscosity, hot paste viscosity, disintegration value, cold glue viscosity, and recovery value of rice. Phosphorus application has been shown to improve the nutritional value of the rice (21). Zhang et al. (22) reported a negative correlation between phosphorus application rate and chalky grain rate, likely due to phosphorus facilitating the transportation of substances in the panicles, resulting in a decrease in chalky grain rate in rice. Chandel et al. (23) reported that increasing the application of phosphorus fertilizer can inhibit the accumulation of amylose in grains. Song et al. (18) reported that the amount of phosphorus fertilizer applied had no significant effect on the amylose content in rice. Although there are published studies of the relationship between nitrogen and phosphorus fertilizer application and rice quality, there are as yet no reports on the differences in the response of rice amylose content and cooking quality to nitrogen–phosphorus interaction regulation, and there are few reports on the effects of nitrogen–phosphorus fertilizer application on the physicochemical structure and properties of rice with different levels of nitrogen efficiency.

Significant differences exist in the response characteristics of nitrogen and phosphorus application among rice varieties with different nitrogen efficiency types (24). Varieties of *Japonica* rice have higher nitrogen utilization efficiency than that in varieties of *Indica* rice (25), and hybrid rice has higher nitrogen utilization efficiency and absorption efficiency than those in the non-hybrid varieties. High-yield varieties have higher nitrogen absorption efficiency and grain nitrogen production efficiency than those of general varieties. The results of numerous studies have shown that nitrogen-efficient rice varieties have higher nitrogen metabolism enzyme activity, stronger root activity, more white roots, a larger root volume, a greater effective absorption area, and a stronger root oxidation capacity than that of nitrogen-inefficient varieties (26, 27). However, further research is needed to determine whether there is a correlation between rice grain quality and the differences in nitrogen and phosphorus application among rice varieties with different nitrogen efficiency types. Accordingly, this study aimed to systematically analyze the effects of different nitrogen and phosphorus fertilizer levels on rice grain quality, starch properties, and structure among rice varieties with differing nitrogen efficiency. In this study, we explored the response mechanisms of starch characteristics and rice quality of different nitrogen-efficient rice varieties to nitrogen and phosphorus application, providing a theoretical basis and technical support for high-yield and high-quality production of rice of different fertilizer response types.

## 2 Materials and methods

### 2.1 Summary of test materials and test sites

The experiment was conducted from 2018 to 2019 at the Agricultural Park Experimental Base of Southwest University of Science and Technology (31°32′N, 104°41′E). The terrain of the experimental site was flat, the soil was loamy, and the baseline fertility level of the experimental field was 1.98 g/kg of total nitrogen, 76.2 mg/kg of available potassium, 43.3 mg/kg of available phosphorus, and 80.3 mg/kg of available nitrogen. The experimental varieties were hybrid rice varieties of different nitrogen efficiency types, obtained through preliminary screening: ‘Deyou 4727’ (low-nitrogen-efficiency type, higher yield under low nitrogen conditions) and ‘Jingyou 781’ (high-nitrogen-efficiency type, higher yield under sufficient nitrogen fertilizer). The previous crop grown on the site was rapeseed under normal fertilization treatment.

### 2.2 Experimental design

A split-block design was adopted, in which the treatment was considered the main zone and the variety the sub-zone. Four different nitrogen application treatments were implemented, as follows: N0 (0 kg/ha), N1 (120 kg/ha), N2 (180 kg/ha), and N3 (240 kg/ha). The phosphorus fertilizer treatments were: P0 (0 kg/ha), P1 (60 kg/ha), P2 (120 kg/ha), and P3 (180 kg/ha). The application rate of potassium fertilizer was 120 kg/ha. Nitrogen was applied at a ratio of 5:3:2 as a base fertilizer and as a fertilizer at the tillering and panicle stages. Phosphorus all used was applied as a base fertilizer. Potash fertilizer was applied as base fertilizer and spike fertilizer at the rate of 1:1, respectively. In total, there were 16 treatments. The experimental design and layout of the plots are shown in Supplementary Figure 1. Each plot was separated by a 50 cm-high and 30 cm-thick cement embankment to prevent seepage of fertilizer or water. The 2-year sowing period was between April 10th and 15th, and the planting period was between May 8th and 12th. Each experimental area was 7 m-long × 7 m-wide, with a planting specification of 33.3 cm × 16.7 cm, and each treatment group consisted of 10 rows × 15 holes/row, with three replications. Other field management was the same as conventional cultivation management. After 3 months of natural drying, the brown rice was sieved through a 1.9 grading sieve and then processed into 90% refined rice by a fully automatic precision machine, and then crushed and sieved (100 mesh) for quality analysis.

### 2.3 Measurement indicators and methods

#### 2.3.1 Determination of appearance and taste value

A rice appearance quality tester (SC-E, Wseen, China) was used to measure the appearance quality indicators of the rice, namely grain length (cm), grain width (cm), length-width ratio (%), chalkiness (%), and chalkiness percentage (%). A rice taste meter (STA1B, SATAKE, Japan), based on optical principles, was used to quantify the taste value of the rice based on appearance, hardness, viscosity, and balance scores. The measurement was repeated three times. Appearance and taste evaluation is the optical evaluation of white rice samples by the Rice Taste Meter, which can be used to judge the characteristics of rice and whether it is well cooked or not, with a full score of 10 points, 8.0 points or more, 8.0 to 7.0 points, slightly better; 7.0 to 6.0 points, average; 6.0 to 5.0 points, slightly worse; and 5.0 points or less, not good. Taste value is the comprehensive evaluation value of rice samples through the rice taste meter, which is a comprehensive score combining the taste and appearance of white rice with the evaluation of functional tests, out of 100 points. Flavor score of 80 points or more, good; 80 to 70 points, slightly better; 70 to 60 points, general; 60 to 50 points, slightly worse; and 50 points or less, bad. The final evaluation of the flavor quality of the samples was based on the comprehensive flavor value for evaluation and ranking.

#### 2.3.2 Determination of amylose content

The amylose content was determined according to the agricultural industry standard NY/T2639-2014 of China.

#### 2.3.3 Thermodynamic properties of starch

Following the method described by Hu et al. (28), a differential scanning calorimeter (DSC 214, Netzsch, Germany) was used to measure the thermal properties of the samples. A 5.00-mg sample of rice flour was placed in an aluminum crucible, to which was added 10 μl of ultra-pure water. The mixture was pressed, sealed, and placed in a refrigerator at 4°C overnight. Before testing, the chilled crucible containing the sample was removed and allowed to stand at room temperature for 1 h. A blank crucible was used as a control. The heating rate of the DSC 214 instrument was set to 10°C/min, with a temperature range of 30–100°C. The thermal effect curve was analyzed using supporting auxiliary software to obtain the starting temperature (To), peak temperature (Tp), termination temperature (Tc), and gelatinization enthalpy. The samples were then stored in a refrigerator at 4°C for 7 days, and the retrogradation characteristics were analyzed. The thermal properties were measured again, using the same procedure as described above. The enthalpy of regeneration was obtained, and the regeneration rate (R) was calculated as: Regeneration rate (R) = regeneration enthalpy / gelatinization enthalpy × 100.

#### 2.3.4 Extraction of starch granules and observation of starch granule morphology

The extraction procedure followed the method of Hu et al. (28), with slight modifications. Weigh 20.00 g of brown rice soaked in water at room temperature overnight, after which it was thoroughly ground in a mortar. The homogenate was loaded into four layers of a gauze bag. The filtrate was squeezed out, and the filtrate was sieved through 100-mesh screens. The filtrate products consisted of starch granules and proteins. The filtrate was centrifuged at 3,000 rpm for 5 min to obtain the precipitate. The sediment was suspended in double-distilled water at 3,000 rpm and centrifuge for 5 min. Sedimentation, suspension, and centrifugation were repeated five times until the supernatant was free of impurities, indicating that proteins in the filtrate were completely removed. Lastly, the product was washed two times with anhydrous ethanol, dried at 40°C for 2 days, ground into starch powder, passed through a 100-mesh sieve, and stored at −20°C for later use.

After the starch granules were extracted, a thin layer was evenly applied to one side of specialized conductive double-sided tape, which was then attached to the lead alloy stage for scanning electron microscopy and sprayed with gold (SCD005 Sputter Coater, Bal-Tec, Pfäffikon, Switzerland). Starch samples were examined using a scanning electron microscope. The magnification of the scanning electron microscope was 5,000 ×, the voltage was 20 KV, and the working distance was 10 mm. Three identical brown rice grains from each variety were gently tapped in the middle with a single-sided blade to break them horizontally in their natural state (perpendicular to the long axis of the rice grains), stuck onto the sample stage with double-sided tape, sprayed with a gold coating on an ion sputtering instrument, and examined using a scanning electron microscope.

#### 2.3.5 Starch crystal structure

An X-ray diffractometer (DMax.B, Rigaku, Japan) was used, with Cu as the target and a graphite monochromator, an accelerated voltage of 30 kV, a current of 20.0 mA, and a step size of 0.02°/2θ. The scanning speed was 0.04°/min, and the scanning range was 3°–40°. The experimental results were analyzed for relative crystallinity using Jade6.0 software. Relative crystallinity (%) was calculated as Ic / (Ia + Ic) × 100, where Ia is the area of the amorphous region in the X-ray diffraction pattern and Ic is the crystallization area.

#### 2.3.6 Data analysis and plotting

Data were analyzed by using analysis of variance (ANOVA), and the means were compared based on the least significant difference (LSD) test at the 0.05 probability level by using SPSS 25.0 (Statistical Product and Service Solutions Inc., Chicago, IL, USA). Origin Pro 2022 (OriginLab, Northampton, MA, USA) was used to draw the figures.

## 3 Results and analysis

### 3.1 Response of quality parameters to nitrogen and phosphorus regulation

**FIGURE 1:**
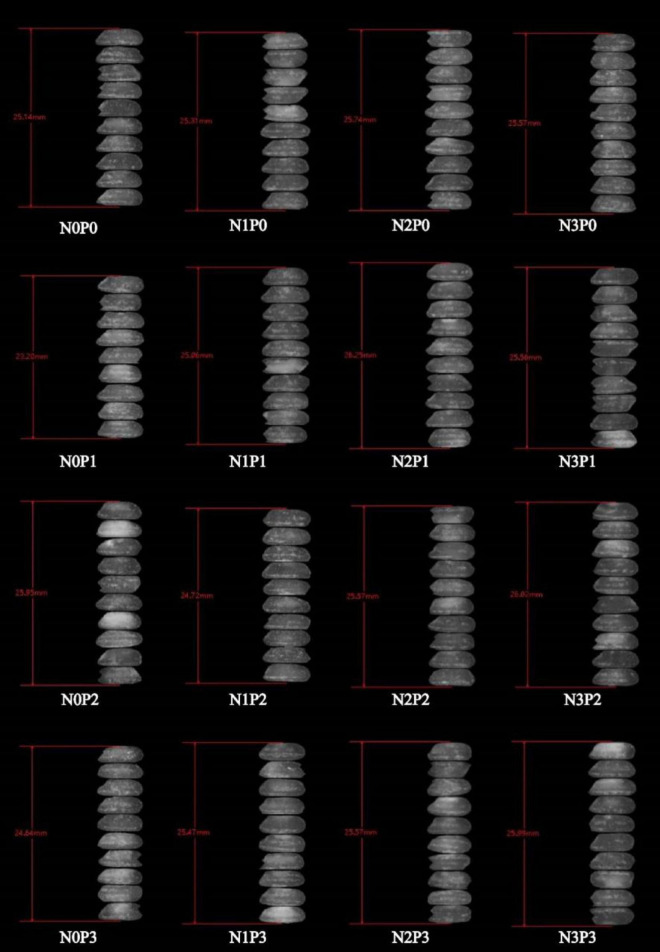
Response of appearance quality of Deyou 4727 to nitrogen and phosphorus.

**FIGURE 2:**
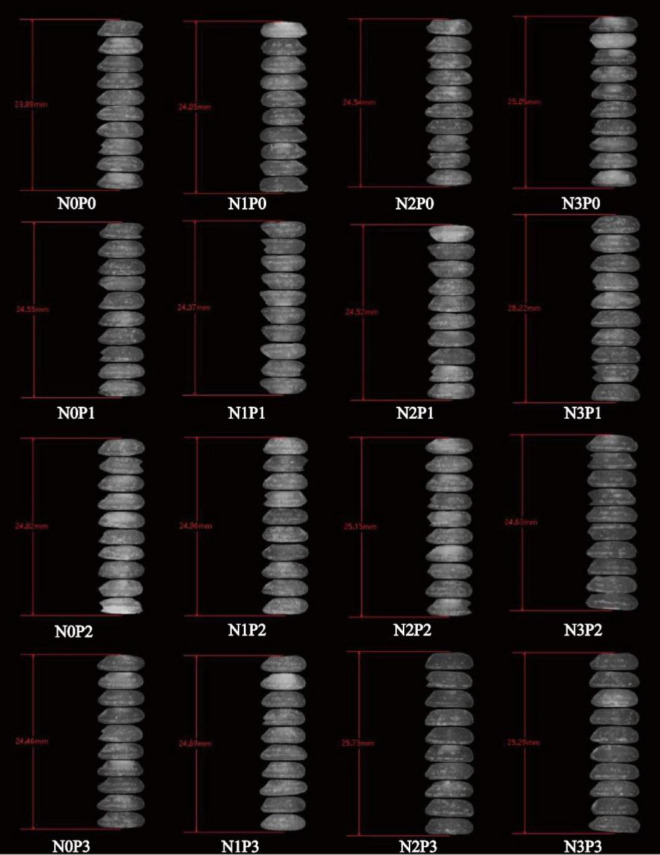
Response of appearance quality of Jingyou 781 to nitrogen and phosphorus. Spots and opaqueness distribution indicate chalkiness; higher spot intensity and more opacity distribution indicate higher chalkiness.

Under the same phosphorus level treatment, an increase in nitrogen fertilizer application resulted in an initial decrease in the chalkiness and chalkiness grain rate of Deyou 4727, followed by an increase (Supplementary Table 1). The length of rice grains did not change significantly (Figure 1). At the N1 level, the chalkiness and grain chalkiness rates of Deyou 4727 were significantly lower than those of the rice groups under the other nitrogen level treatments, which indicates improved appearance. After increasing the amount of nitrogen fertilizer, the chalkiness and chalky grain rate of Jingyou 781 decreased (Supplementary Table 1). The length of the rice grains changed less, and the width of the grains was significantly affected by the nitrogen–phosphorus interaction (Figure 2). The high nitrogen (N3) treatment significantly improved the appearance and quality of high nitrogen-efficient rice. At the same nitrogen level, the application of phosphorus fertilizer significantly reduced the chalkiness of Deyou 4727, and the chalkiness rate showed a trend of first decreasing and then increasing with increasing phosphorus fertilizer application. Under the P2 conditions, the chalkiness and chalkiness rates reached their lowest values. Increasing the application of phosphorus fertilizer significantly reduced the chalkiness and chalky grain rate of Jingyou 781, which showed the highest appearance quality under P3-level treatment.

Nitrogen, phosphorus, and nitrogen–phosphorus interaction effects all had a significant impact on the appearance of Deyou 4727. Under different nitrogen levels, with an increase in phosphorus fertilizer application, the chalkiness and chalky grain rate of Deyou 4727 showed a trend of first decreasing and then increasing, reaching the lowest values under P1 or P2 conditions. However, the value for Jingyou 781 decreased significantly with increasing phosphorus application. In addition, the response of Jingyou 781 rice grain width to nitrogen showed that under P0 and P1 conditions, the rice grain width increased with an increase in nitrogen fertilizer application and showed a trend of first increasing and then decreasing under P2 and P3 conditions (Figure 2).

### 3.2 Taste value and apparent amylose content ANOVA

Supplementary Table 2 shows that the apparent amylose content of hybrid rice is highly significantly affected by varieties, nitrogen and phosphorus and their interactions, and there are highly significant differences in taste values between varieties, the effects of nitrogen fertilizer and nitrogen fertilizer and the interactions between varieties on taste values reach highly significant levels, and phosphorus fertilizer treatments do not have a significant effect on taste values.

#### 3.2.1 Response of eating quality to nitrogen and phosphorus application

**FIGURE 3:**
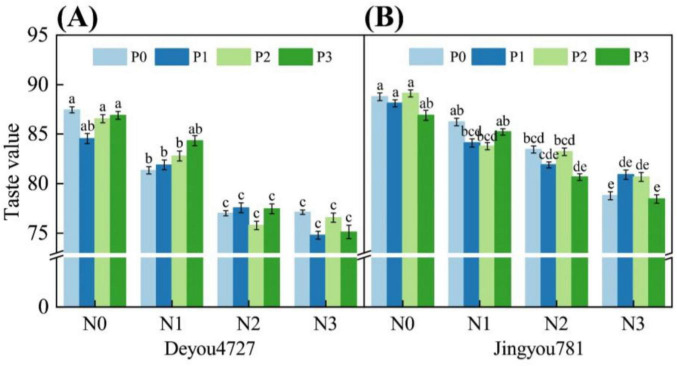
The effect of nitrogen and phosphorus application on the eating value of rice. Different letters within the same treatment in the figure indicate significant differences at the *P* = 0.05 level. **(A)** Deyou4727, **(B)** Jingyou781.

As shown in Figure 3, the single application of nitrogen and phosphorus and their interactions had a significant effect on the food value (palatability) of Deyou 4727 and Jingyou 781. The food value of Deyou 4727 reached its maximum value (87.44) under the N0P0 condition and the lowest value (74.78) under the N3P1 condition. The food value of Deyou 4727 showed a decreasing trend after increasing nitrogen fertilizer application, and there was a significant difference between the N1 and N2 levels. The food quality of Jingyou 781 reached its maximum (89.11) under N0P2 conditions and its lowest (78.44) under N3P3 conditions. After adding nitrogen fertilizer, the food value of Jingyou 781 showed a significant decrease trend and was significantly reduced by 3.84% (N1), 6.71% (N2), and 9.67% (N3) compared with the nitrogen-free (N0) treatment. The application of phosphorus fertilizer had no significant effect on the food values of Deyou 4727 and Jingyou 781. The effect of nitrogen–phosphorus interaction regulation on the food quality of Jingyou 781 was greater than that of Deyou 4727. The food quality of Deyou 4727 was lower than that of Jingyou 781, and the food value of Deyou 4727 was more affected by the adverse effects of increasing nitrogen fertilizer than that of Jingyou 781.

#### 3.2.2 Apparent amylose

**FIGURE 4:**
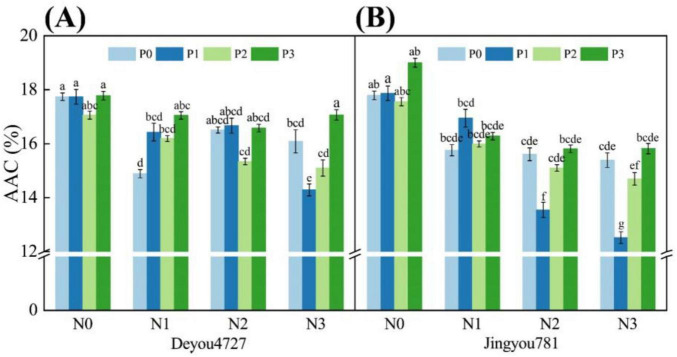
The effect of nitrogen and phosphorus application on the content of amylose in rice. Different letters within the same treatment in the figure indicate significant differences at the *P* = 0.05 level. **(A)** Deyou4727, **(B)** Jingyou781.

As shown in Figure 4, under the conditions of nitrogen and phosphorus and their interactions, the apparent amylose content (AAC) of Deyou 4727 and Jingyou 781 changed significantly. The AAC of Deyou 4727 ranged from 14.00% to 18.00%. After increasing the nitrogen fertilizer application, the AAC showed a decreasing trend. Under the nitrogen–phosphorus interaction treatment, increasing the application of phosphorus fertilizer under N0, N2, and N3 conditions resulted in a decreasing and then increasing trend in the AAC in Deyou 4727. Under N1 conditions, increasing the application of phosphorus fertilizer resulted in an increasing trend in AAC of Jingyou 781, ranging from 12.00% to 19.00%, and this change was significantly greater than that of Deyou 4727. The application of the nitrogen fertilizer Jingyou 781 showed a decreasing trend in AAC. Under the nitrogen–phosphorus interaction treatment, increasing the application of phosphorus fertilizer under N0 conditions increased the AAC of Jingyou 781, whereas, under N1 conditions, the AAC value of the application of phosphorus fertilizer first increased and then decreased. Under N2 and N3 conditions, there was a trend of AAC first decreasing and then increasing.

### 3.3 Thermodynamic properties of starch

As shown in Supplementary Table 3, increased nitrogen fertilizer application significantly reduced the starch thermal parameters (onset temperature, To; peak temperature, Tp; and conclusion temperature, Tc) and gelatinization enthalpy (ΔHg) of Deyou 4727 and significantly increased the starch retrogradation enthalpy (ΔHr) and retrogradation rate (R). In the case of Deyou 4727, with the increase in phosphorus fertilizer application, ΔHg showed a trend of first increasing and then decreasing. ΔHr and R showed an initial increase, followed by a decrease and then an increase, both reaching their maximum values under P3 conditions. The effect of phosphorus fertilizer on the starch retrogradation characteristics of Deyou 4727 was significantly higher than that of nitrogen fertilizer treatment. ΔHr and R first increased and then decreased with the increase in phosphorus fertilizer application under nitrogen application conditions. Under low to medium nitrogen conditions, P2 was observed to reduce ΔHr and R of Deyou 4727, ultimately resulting in rice with better food quality (Supplementary Table 3-1).

In the case of Jingyou 781, after nitrogen application, ΔHg was the lowest under N2 conditions and ΔHr was the lowest under N3 conditions. The R-value under N2 was lower than that of the N0 treatment. Under the condition of applying phosphorus fertilizer alone, Tp was the lowest at P2 and the highest at P1. R was the lowest at P1 and the highest at P2. When nitrogen fertilizer was applied alone, Tp was the lowest under N1 and highest under N2, whereas R was the lowest under N3 and highest under N2. The regulation of the nitrogen–phosphorus interaction had a significant impact on the thermodynamic properties of Jingyou 781 starch. Applying phosphorus fertilizer under N1 significantly increased To, whereas increasing phosphorus fertilizer under N2 significantly reduced To. Applying phosphorus fertilizer under N3 conditions was observed to increase ΔHg (Supplementary Table 3-2). With the increase in nitrogen fertilizer application, the low phosphorus treatment significantly increased ΔHr and R, and under the N3, P3 treatment, ΔHr and R were significantly higher than other treatment combinations. For low-nitrogen and high-efficiency rice, the interaction between medium-high nitrogen and low-medium phosphorus can significantly reduce Tp and R, ultimately improving the food quality of rice.

### 3.4 Starch crystal structure

**FIGURE 5:**
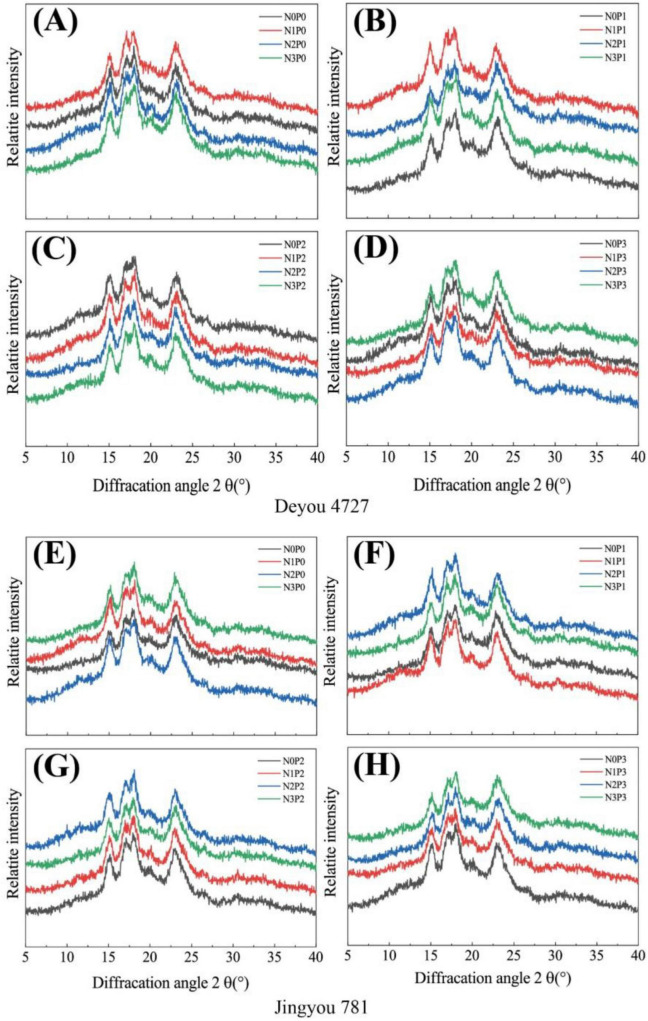
Wide angle X-ray diffraction pattern (2θ) of Deyou 4727 and Jingyou 781 under nitrogen and phosphorus treatment. **(A–D)** Wide-angle X-diffraction patterns of Dewei 4727 under the same phosphorus treatment and different nitrogen treatments; **(E–H)** wide-angle X-diffraction patterns of SPARKLE 781 under the same phosphorus treatment and different nitrogen treatments.

The X-ray diffraction patterns of starch Deyou 4727 and Jingyou 781 are shown in Figure 5. At 2θ diffraction angles of 15° and 23°, there were two strong peaks, and at 17° and 18°, there were continuous double peaks, which are typical of A-type starch crystals. Referring to Supplementary Table 4, under phosphorus-free or low phosphorus treatments (P0 and P1), increasing nitrogen fertilizer resulted in a trend of first increasing and then decreasing the starch crystallinity of Deyou 4727, whereas, under high phosphorus treatment (P3), the starch crystallinity first decreased and then increased. Under N0 treatment, increasing the application of phosphorus fertilizer led to a trend of first decreasing and then increasing the starch crystallinity of Deyou 4727. Under N1, N2, and N3 treatments, increasing the application of phosphorus fertilizer resulted in an increase followed by a decrease in the starch crystallinity of Deyou 4727. Under high phosphorus treatments (P2 and P3), increasing nitrogen fertilizer resulted in an increasing trend in the crystallinity of Jingyou 781 starch (Supplementary Table 4). In addition, under the same nitrogen treatment, the application of high-phosphorus fertilizer (P2 and P3) significantly increased the starch crystallinity of Jingyou 781 compared to that of P0 and P1. The response of the starch crystallinity of Deyou 4727 to nitrogen fertilizer application was weaker than that of Jingyou 781. Under low nitrogen (N1) conditions, the crystallinity of Deyou 4727 reached its highest value; further increasing nitrogen fertilizer application led to a decrease in crystallinity. The crystallinity of Jingyou 781 starch was highest under high-nitrogen (N3) conditions and was lower than Deyou 4727 under nitrogen-free (N0) conditions.

### 3.5 Endodermal tissue arrangement

**FIGURE 6:**
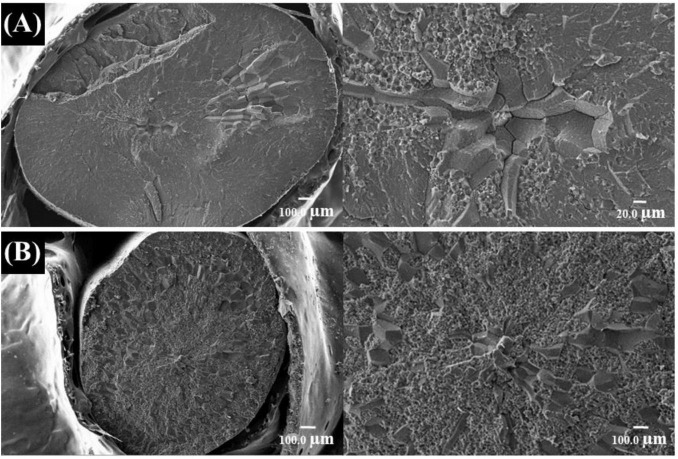
Cross-sections of low- **(A)** and high-chalky grains **(B)**.

Different rice varieties exhibited high and low chalkiness under N–P interaction conditions. The typical arrangement and morphology of endosperm cells in the cross-sections of low- and high-chalky grains are shown in Figure 6. The arrangement pattern of endosperm cells in the cross-section of the tested brown rice was consistent. Endosperm cells radiate from or near the center as a concentric circle, radiating outward from the center. The endosperm cells in the central position were relatively small compared with the outer layer cells, which is consistent with the results reported by other researchers. The cross-section of the low-chalky white grains was smooth, with tightly arranged endosperm cells and few gaps between cells. The endosperm cells of the larger chalky grains were arranged loosely with larger cell gaps, and the cross-section of the grains was powdery. Both high- and low-chalkiness grains had a loose arrangement of endosperm cells in the powdery area, but low-chalkiness grains had fewer powdery areas and fewer endosperm cell gaps, resulting in high transparency and low chalkiness. The main reason for this was the large amount of air space between the rice endosperm cells, which diffused light and made the kernel appear opaque, consistent with previous research findings.

**FIGURE 7:**
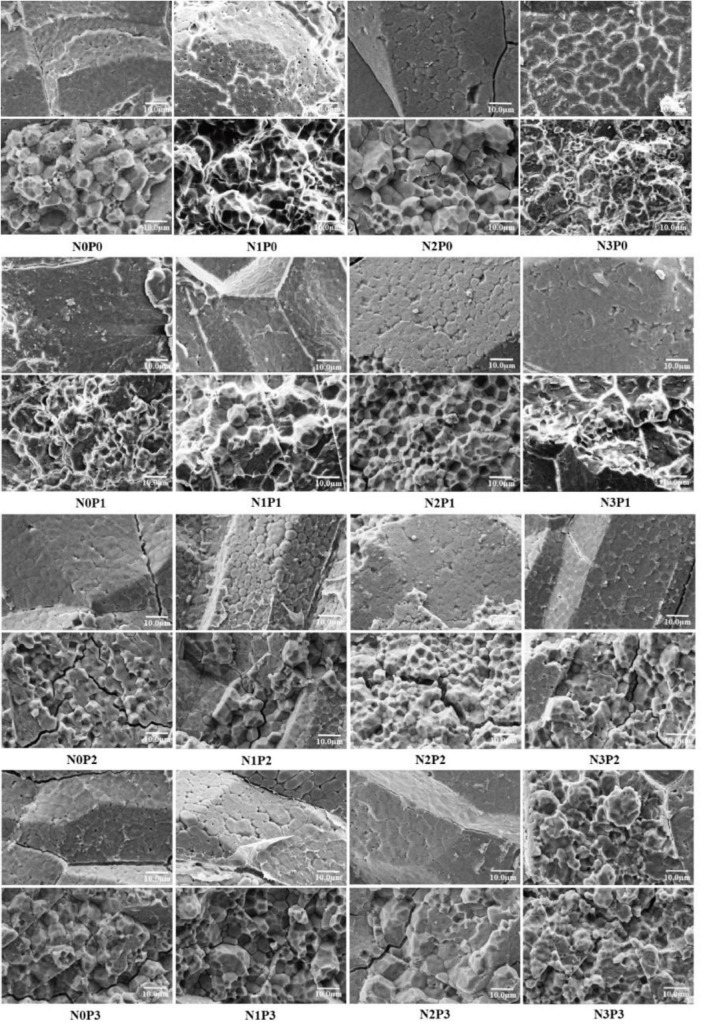
Cross section scanning electron microscope of Deyou 4727 under nitrogen and phosphorus treatment.

**FIGURE 8:**
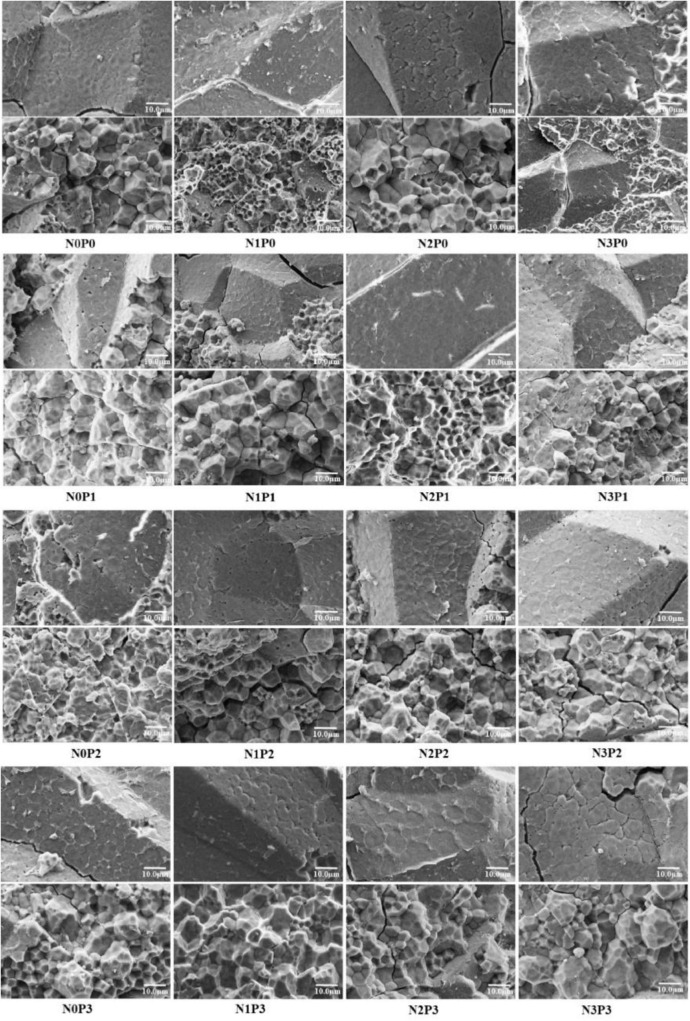
Scanning electron microscopy of the cross-section of Jingyou 781 rice under nitrogen and phosphorus treatment.

Representative grains of different types of rice varieties under nitrogen–phosphorus interaction treatment were selected for cross-sectional scanning, and the differences between the dense and powdery regions were compared, as shown in Figures 7, 8. With increasing phosphorus fertilizer application, the chalky grain rates of Jingyou 781 and Deyou 4727 decreased (Figures 1, 2 and Supplementary Table 2), the powdery area of the grain cross-section decreased, and the compactness of the endosperm cell arrangement increased. With an increase in phosphorus fertilizer application in the powdery area, cell wall adhesion between endosperm cells increased, and the proportion of smooth, round cell walls resembling endosperm cells decreased, resulting in tighter cell adhesion. With increasing nitrogen fertilizer application, the powdered area of the grain cross-section of Jingyou 781 decreased, and the arrangement of endosperm cells became more compact. As for Deyou 4727, the application of nitrogen fertilizer increased the arrangement density of its endosperm cells; however, under N3 conditions, there was an increasing trend in the powdery area. Under different phosphorus application conditions, the N2 treatment had the highest arrangement density of grain endosperm cells, and its chalkiness and chalky grain rate reached the lowest values under N2 conditions.

### 3.6 Starch particle morphology and size

**FIGURE 9:**
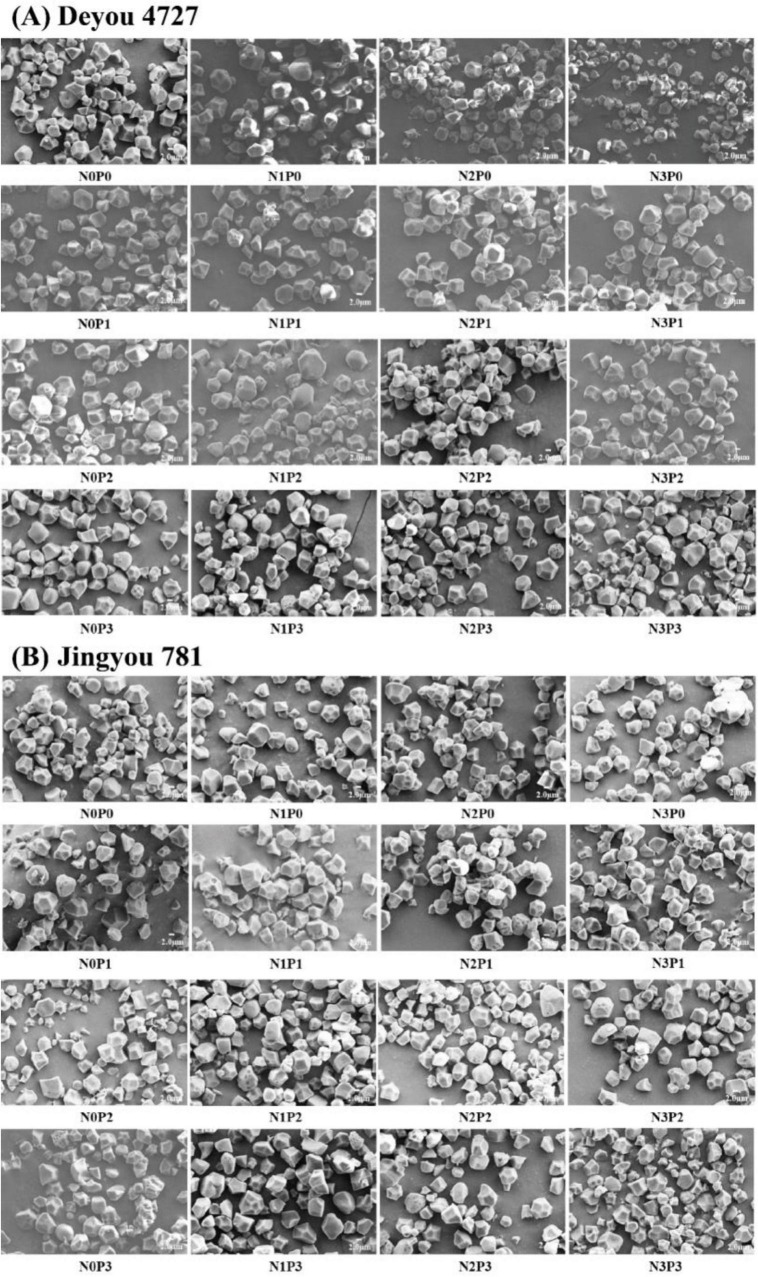
Scanning electron microscope of starch grains of Deyou 4727 **(A)** and Jingyou 781 **(B)** under nitrogen and phosphorus treatment.

The starch particle size of two rice varieties ranged from 1 to 10 μm. Most particle sizes range from 2 to 5 μm (Figure 9 and Supplementary Tables 5-1, 5-2). Most starch granules have a regular polyhedral shape with flat edges, whereas a few have smooth, spherical, or ellipsoidal edges. Significant differences exist in starch grain size and shape among the different rice varieties and nitrogen–phosphorus interaction treatments.

Increased application of nitrogen fertilizer improved the proportion of medium-sized (2–5 μm), small-sized (<2 μm), and polyhedral starch particles of Deyou 4727, with a corresponding reduction in the ratio of large-sized (>5 μm), spherical, or ellipsoidal starch granules. The proportions of medium-sized and conventional polyhedral starch granules increased in Jingyou 781, with a corresponding reduction in the proportion of small, large, spherical, and ellipsoidal starch granules. The application of phosphorus fertilizer increased the proportion of medium-sized, conventional polyhedral starch granules in Deyou 4727 and reduced the proportion of small-sized, large-sized, spherical, or ellipsoidal starch granules. As for Jingyou 781, the increase in phosphorus increased the proportion of small-sized and spherical or elliptical starch granules and reduced the proportion of large and conventional polyhedral starch granules.

Significant differences exist in starch grain size and shape among the different rice varieties under the nitrogen–phosphorus interaction treatment. Under the N0 and N3 conditions, increasing the application of phosphorus fertilizer in Deyou 4727 resulted in a trend of first decreasing and then increasing the proportion of small starch particles. In the low- and high-efficiency rice varieties, as the nitrogen application rate increased, the proportion of medium-sized starch particles in Deyou 4727 showed a trend of first increasing and then decreasing. A single application of phosphorus fertilizer increased the proportion of large-sized starch particles, whereas under nitrogen application conditions, increasing the application of phosphorus fertilizer significantly reduced the proportion of large-sized starch particles. Applying phosphorus fertilizer under N1 and N2 significantly reduced the proportion of spherical or ellipsoidal starch particles while increasing the proportion of conventional polyhedral starch particles. For the high-nitrogen and high-efficiency rice varieties, adding conventional phosphorus fertilizer under N0 resulted in a decrease in the proportion of starch particles in Jingyou 781; under various nitrogen application conditions, the proportion of large starch particles in the application of phosphorus fertilizer in Jingyou 781 showed a trend of first decreasing and then increasing. Under N1 and N2 conditions, the application of phosphorus fertilizer significantly increased the proportion of spherical or ellipsoidal starch particles; and under N3 conditions, the proportion of spherical or ellipsoidal starch granules gradually decreased with the application of phosphorus fertilizer, whereas the proportion of conventional polyhedral starch changed.

### 3.7 Correlation of nitrogen and phosphorus levels on food quality, starch grain structure, and crystallization

A significant correlation exists between nitrogen and phosphorus application rates and the food quality, starch grain type and structure, and starch crystallization characteristics of rice. These correlations varied significantly among the varieties (Supplementary Tables 6, 7).

The application of nitrogen fertilizer reduced the chalkiness of Deyou 4727, whereas phosphorus fertilizer reduced the chalkiness rate. At the same time, nitrogen and phosphorus applications increased the proportion of small-sized starch particles and decreased that of large-sized starch particles (Supplementary Table 6). The amount of nitrogen fertilizer applied was significantly negatively correlated with chalkiness and the proportion of large-sized starch particles in Deyou 4727, whereas it was significantly positively correlated with the proportion of small-sized starch particles. The amount of phosphorus fertilizer was significantly positively correlated with the chalky grain rate and proportion of medium-sized starch particles, whereas it was significantly negatively correlated with the proportion of large-sized starch particles. Chalkiness characteristics were positively correlated with the proportion of medium-sized starch particles and negatively correlated with the proportion of large starch particles. Chalkiness showed a highly significant negative correlation with the proportion of small starch particles and a highly significant positive correlation with the proportion of large starch particles. Chalkiness was significantly and positively correlated with the proportion of round starch granules and significantly and negatively correlated with the proportion of regular starch granules.

A positive correlation exists between nitrogen and phosphorus application and starch crystallinity in Jingyou 781 and a highly significant positive correlation between nitrogen fertilizer application and starch crystallinity. The opposite correlation was observed between the amount of nitrogen and phosphorus fertilizers applied and the distribution of starch particle size in Jingyou 781. The amount of nitrogen fertilizer applied was significantly negatively correlated with the proportion of small-sized starch particles, significantly positively correlated with the proportion of medium-sized starch particles, and significantly negatively correlated with the proportion of phosphorus fertilizer and medium-sized starch particles. The amount of nitrogen fertilizer applied showed a highly significant negative correlation with the proportion of round starch granules in Jingyou 781, whereas the correlation with the proportion of regular starch granules was the opposite. The correlation between phosphorus fertilizer application and the shape of the Jingyou 781 starch granules was not significant. The chalkiness of Jingyou 781 was significantly negatively correlated with starch crystallinity. Chalkiness was significantly positively correlated with the proportion of small- and large-sized starch particles and significantly negatively correlated with the proportion of medium-sized starch particles. Chalkiness was significantly and positively correlated with the proportion of round starch granules and significantly and negatively correlated with the proportion of regular starch granules.

### 3.8 Differences in yield response to nitrogen and phosphorus application

**FIGURE 10:**
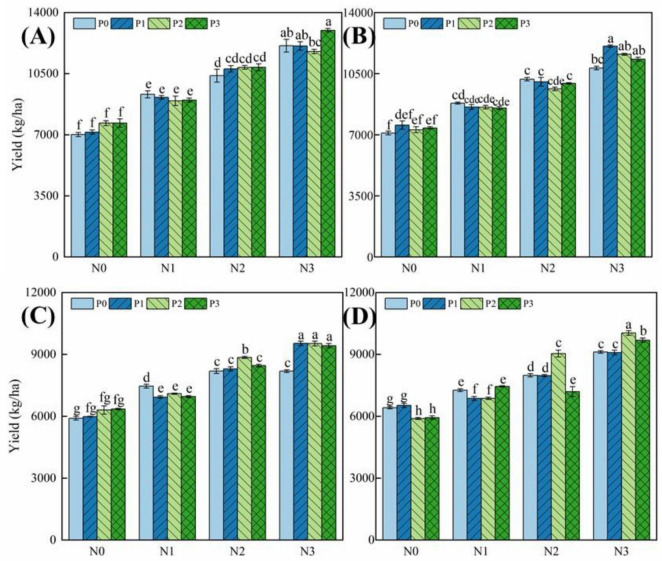
Effect of nitrogen and phosphorus application on hybrid rice yield (2018 and 2019). Panels **(A,B)** represent the yields of Deyou 4727 and Jingyou 781 under nitrogen and phosphorus treatments in 2018, respectively; panels **(C,D)** represent the yields of Deyou 4727 and Jingyou 781 under nitrogen and phosphorus treatments in 2019, respectively; Different letters within the same treatment in the figure indicate significant differences at the *P* = 0.05 level.

As can be seen in Figure 10, the yield of different hybrid rice after continuous nitrogen and phosphorus treatments increased significantly with the increase in nitrogen fertilizer application; the effect of phosphorus fertilizer on hybrid rice yield is relatively small, there is a certain inhibitory effect of excessive application of nitrogen on the yield, and phosphorus fertilizer mainly affects hybrid rice yield through the interactions with nitrogen fertilizer effects. The yields of different nitrogen-efficient rice varieties did not differ much at different levels of nitrogen fertilizer, but there were significant differences in the response of the yields of different nitrogen-efficient rice varieties to nitrogen fertilizer, and the yield-increasing effect of Deyou 4727 was higher than that of Jingyou 781 after additional nitrogen fertilizer application. The yield-increasing effect of Deyou 4727 was higher than that of Jingyou 781 in the medium and low nitrogen (N1 and N2) conditions after the application of nitrogen fertilizer by 5.71% (N1) and 9.77% (N2), respectively (2018), and 1.02% (N1) and 7.76% (N2) higher than Jingyou 781 in 2019, respectively. It can be seen that the yield increase effect after nitrogen application of low-nitrogen-high efficiency rice varieties was higher than that of high-nitrogen-high-efficiency rice varieties under medium-low nitrogen conditions; however, the yield increase effect of high-nitrogen-high-efficiency rice varieties was greater than that of low-nitrogen-high efficiency rice varieties after successive excess application of nitrogen, and Jingyou 781 had a higher yield increase effect than Deyou 4727 after successive high-nitrogen (N3) treatments by 3.76%, and the average yield under high-nitrogen was also higher than that of Deyou 4727 by increased by 3.46% (2019).

## 4 Discussion

### 4.1 Differences in response to quality traits of rice with different nitrogen efficiency types

Nitrogen is an important nutrient for increasing grain yield and protein content, and excess nitrogen can reduce grain quality, especially in terms of food quality (29). Optimal nitrogen fertilizer application increases rice yield and improves rice quality (30, 31). Regarding the effect of phosphorus application on rice quality, previous studies have suggested that the application of phosphorus fertilizer can significantly reduce the chalkiness rate and chalkiness degree and improve the nutritional and eating quality of rice (32). In one study, the amylose content and taste value of rice were significantly decreased with increases in the nitrogen application level (33). In this experiment, it was found that low nitrogen and phosphorus had higher palatability values, and the quality traits of hybrid rice (appearance, food quality, starch thermodynamic properties, endosperm cell arrangement, and starch particle size) were significantly influenced by variety, nitrogen–phosphorus interactions, and their interaction effects. The impact of nitrogen fertilizer treatment on quality was greater than that of phosphorus fertilizer treatment. The influence of various factors on appearance reached a highly significant level, which was related to the quality differences between the two varieties. The response of the appearance quality of rice varieties with different nitrogen efficiencies to nitrogen fertilizer was different. Low nitrogen efficiency rice varieties showed a trend of first decreasing and then increasing chalkiness and grain chalkiness rate with an increase in nitrogen fertilizer application, whereas high nitrogen efficiency rice varieties showed a decreasing trend in appearance quality with an increase in nitrogen fertilizer application. Under high nitrogen (N3) conditions, the appearance quality of high nitrogen-efficient rice varieties could be significantly improved, while low nitrogen-efficient rice varieties require relatively low nitrogen fertilizer application levels to maintain high appearance quality.

### 4.2 Changes in starch properties and structure under nitrogen and phosphorus application

The gelatinization process of starch involves the breaking and decomposition of the double-helical structure of branched starch in starch crystals. Differences in the arrangement, compactness, and shape of starch granules, and the amylose content, can cause differences in the leaching rate (34), thereby significantly affecting the gelatinization process of rice. The short-chain segments of amylose and amylopectin in starch molecules form double-helix structures, and these double-helix molecular chains form different polycrystalline forms in certain areas of starch particles through intermolecular interactions in a certain spatial lattice (35). Natural starches can be classified as A-, B-, and C-types based on their XRD patterns. In general, waxy and normal cereal endosperms have A-type starch and tubers; high-cellulose cereal starch has B-type XRD patterns; and legume and yam starches have C-type patterns (36). Therefore, combining the amylose content, starch structure, viscosity characteristics, and crystallization characteristics can reveal the mechanisms by which nitrogen and phosphorus fertilizers affect rice quality.

The thermodynamic characteristic values Tp, △Hg, △Hr, and R of starch in low-nitrogen and high-efficiency rice Deyou 4727 were significantly affected by nitrogen phosphorus and its interaction effects. To, Tp, and Tc are more affected by nitrogen fertilizer effects than phosphorus fertilizer effects, while △Hg, △Hr, and R% were more affected by phosphorus fertilizer effects. The thermodynamic properties of starch in the high-nitrogen and efficient rice variety Jingyou 781 were significantly or extremely significantly affected by nitrogen and phosphorus and their interaction effects. The effect of nitrogen fertilizer on starch To, Tp, Tc, and △Hg was greater than that of phosphorus fertilizer, and R was relatively more affected by the effect of phosphorus fertilizer. In comparison, the response of low nitrogen and high-efficiency rice Tp and △Hr to nitrogen and phosphorus fertilizers was greater than those of high nitrogen and high-efficiency rice. To and Tc were affected more by nitrogen and phosphorus fertilizers in high-nitrogen and high-efficiency rice than in low-nitrogen and high-efficiency rice, and the starch recovery rate (R) was higher in high-nitrogen and high-efficiency rice. In terms of nitrogen–phosphorus interaction, the response of low-nitrogen and high-efficiency rice △Hr to nitrogen–phosphorus interaction was greater than that of high-nitrogen and high-efficiency rice. High-nitrogen and high-efficiency rice To, Tc, Tp, and R were more affected by nitrogen–phosphorus interactions than low-nitrogen and high-efficiency rice, indicating that the thermodynamic characteristics of high-nitrogen and high-efficiency rice were greatly affected by nitrogen and phosphorus. This may be due to the positive effect of nitrogen and phosphorus application on the amylose content of high-nitrogen and high-efficiency rice. Therefore, high-nitrogen-efficient rice had a higher content of amylose, which made the starch gelatinization process easily affected.

Starch characteristics mainly refer to its degree of dissolution, swelling, gelatinization, and cooling regeneration during cooking or heating when in contact with water. A higher amylose content limits starch expansion, while proteins hinder the development of starch mesh-like structures, limiting the ability of starch to absorb water and causing incomplete gelatinization (37). This study observed that the starch particle size of the tested rice varieties ranged from 1 to 10 μm. Most particle sizes ranged from 2 to 5 μm. Starch granules can be divided into regular polyhedral shapes with straight edges and spherical or ellipsoidal shapes with smooth edges. The proportion of starch granules with polyhedral shapes was relatively high; however, with an increase in chalkiness, the proportion of spherical or ellipsoidal starch granules increased. The more uniform the distribution of starch granules, the fewer the layers of long columnar cells, and the less chalkiness, which is consistent with previous research (19, 38). Significant differences exist in starch grain size and morphology among the different rice varieties and nitrogen–phosphorus interaction treatments. Nitrogen–phosphorus interaction treatment can change the starch morphology and crystallization characteristics of rice grains, and there are certain differences in the response of the starch structure to nitrogen and phosphorus in different nitrogen-efficient rice varieties. The addition of nitrogen fertilizer increased the starch crystallinity of rice. The starch crystallinity of low-nitrogen and high-efficiency rice varieties first increased and then decreased with an increase in nitrogen fertilizer application, whereas high-nitrogen and high-efficiency rice varieties always showed an increasing trend with an increase in nitrogen fertilizer application. The application of phosphorus fertilizer showed an increasing trend in starch crystallinity in rice varieties with different nitrogen efficiency types, which was consistent with the response of the chalky appearance characteristics of different rice varieties to nitrogen and phosphorus.

## 5 Conclusion

In this study, we used low- and high-nitrogen-efficient rice varieties to measure the changes in rice quality and differences in starch structure under 16 nitrogen and phosphorus interaction treatment. Increasing the application of nitrogen fertilizer improved the appearance, quality, retrogradation rate, proportion of medium-sized starch particles, and relative crystallinity of the rice grains. Increased nitrogen reduced the amylose content, food quality, starch retrogradation enthalpy, gelatinization enthalpy, and proportion of large starch particles, increased the stability of the starch crystal structure, decreased starch particle size, and hindered starch expansion and gelatinization. Increasing the application of phosphorus fertilizer increased the appearance, quality, gelatinization enthalpy, proportion of medium-sized starch particles, and relative crystallinity of the rice while reducing the food quality, starch retrogradation enthalpy, and proportion of small- and large-sized starch particles. With an increase in nitrogen and phosphorus fertilizer applications, the differences in starch physicochemical properties and structures became more significant. High-nitrogen-efficient rice varieties can significantly improve appearance quality under high nitrogen conditions, and the interactions of medium-high nitrogen and low-medium phosphorus can lead to a significant decrease in starch thermal parameters and retention rate, thus improving rice cooking quality. Low-nitrogen-efficient rice varieties can also improve the quality of rice under low-medium-nitrogen conditions with appropriate application of phosphorus fertilizer.

## Data Availability

The original contributions presented in this study are included in the article/Supplementary material, further inquiries can be directed to the corresponding authors.
