# Supplementary material for: Effect of nitrogen and phosphorus application on starch characteristics and quality of rice with different nitrogen efficiency
Source: Front Nutr. 2024 Oct 22;11:1462689. doi: 10.3389/fnut.2024.1462689 (PMC11534870; doi:10.3389/fnut.2024.1462689)
Supplement: Supplementary file 1 [file Data_Sheet_1.docx]

**
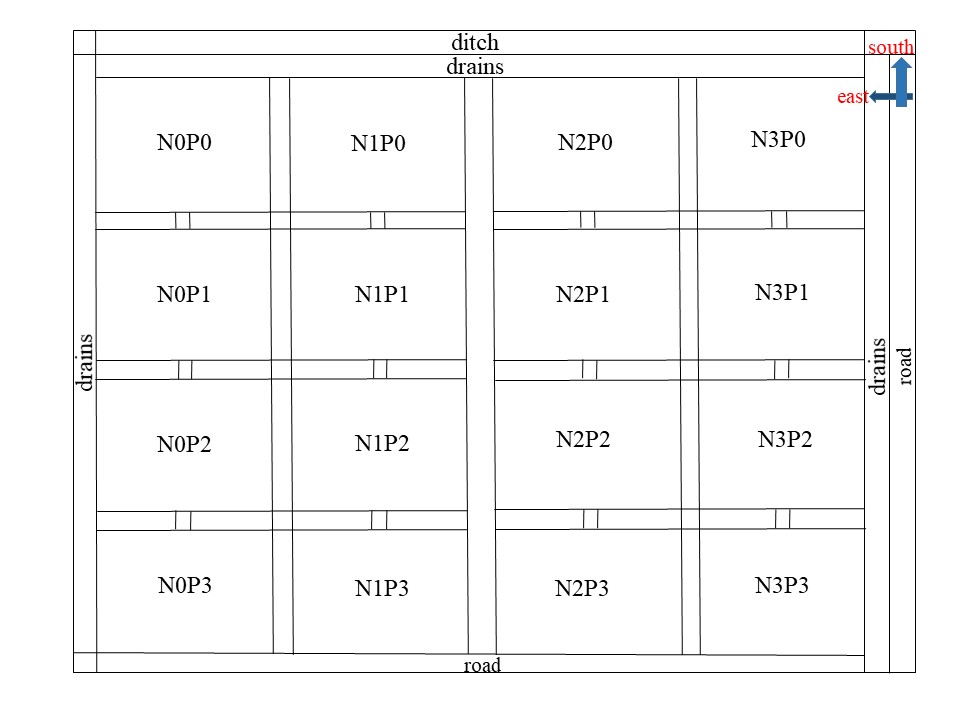
Figure S1.** Coupling treatment of nitrogen and phosphorus in fixed position field

| Treat | | Deyou 4727 | | Jingyou 781 | |
| --- | --- | --- | --- | --- | --- |
|  |  | chalkiness | chalky grain rate | chalkiness | chalky grain rate |
| N0 | P0 | 21.85±0.60b | 32.94±0.02e | 22.96±0.71a | 35.33±0.08a |
|  | P1 | 18.08±0.56d | 28.33±0.17g | 21.11±0.30ab | 32.24±0.05c |
|  | P2 | 17.62±0.21de | 24.12±0.22i | 16.45±0.11e | 32.73±0.02b |
|  | P3 | 13.82±0.15g | 35.00±0.01d | 15.24±0.75f | 28.84±0.24g |
| N1 | P0 | 16.64±0.61ef | 26.14±0.18h | 21.72±1.67ab | 28.52±0.18h |
|  | P1 | 15.87±0.28f | 26.94±0.16h | 20.41±0.72bc | 30.79±0.02e |
|  | P2 | 15.34±0.55f | 22.41±0.24j | 18.53±0.55d | 28.00±0.13i |
|  | P3 | 13.69±0.09g | 24.90±0.36i | 13.07±0.28g | 23.85±j0.03 |
| N2 | P0 | 23.87±0.88a | 26.82±0.08h | 21.94±0.88ab | 35.56±0.04a |
|  | P1 | 18.63±0.15d | 30.44±0.04f | 20.61±0.22bc | 30.00±0.02f |
|  | P2 | 16.22±0.32f | 34.29±0.06d | 19.28±0.62cd | 23.03±0.07l |
|  | P3 | 12.69±0.09g | 40.94±0.07c | 13.71±0.64g | 20.67±0.20n |
| N3 | P0 | 23.97±0.46a | 52.09±0.01a | 20.19±0.94bc | 31.35±0.15d |
|  | P1 | 20.00±0.23c | 46.58±0.13b | 19.43±0.82cd | 23.44±0.22k |
|  | P2 | 16.00±0.90f | 22.00±1.82j | 19.43±1.67cd | 21.38±0.02m |
|  | P3 | 16.39±0.62f | 40.68±0.08c | 11.95±12.95g | 21.28±0.02m |
| F Value | N | 251.85** | 682.96** | 146.27** | 8075.16** |
|  | P | 78.60** | 1427.58** | 19.25** | 5757.78** |
|  | N×P | 48.32** | 460.76** | 1.61ns | 1079.45** |

**Table S1.** Effects of nitrogen and phosphorus regulation on appearance quality of rice

Note: Different letters within the same treatment in the figure indicate significant differences at the P=0.05 level;N and P in the F value are nitrogen application rate and Phosphorus application rate. N×P is the interaction between nitrogen application rate and Phosphorus application rate. * and ** represent significant levels of 0.05 and 0.01, respectively.

**Table S2.** Variance analysis of response of taste value and amylose content to nitrogen and phosphorus regulation

| Source of variation | AAC | Taste Value |
| --- | --- | --- |
| PV | 63.95** | 102.08** |
| P | 17.37** | 1.22ns |
| N | 55.42** | 168.02** |
| PV×P | 8.09** | 2.30ns |
| PV×N | 8.52** | 6.00** |
| N×P | 7.77** | 1.22ns |
| PV×P×N | 9.75** | 1.75ns |

Notes: AAC: the apparent amylose content. PV: variety; P: phosphorus fertilizer treatment; N: nitrogen fertilizer treatment; PV×P: variety and phosphorus fertilizer intercropping; PV×N: variety and nitrogen fertilizer intercropping; N×P:nitrogen-phosphorus intercropping; PV×P×N: variety and nitrogen-phosphorus intercropping. * and ** represent significant levels of 0.05 and 0.01, respectively, ns: indicates no significant level.

**Table S3-1.** Response of Deyou 4727 rice DSC content to nitrogen and phosphorus regulation

| Treat | | To(℃) | Tp(℃) | Tc(℃) | ∆Hg(J/g) | ∆Hr(J/g) | R(%) |
| --- | --- | --- | --- | --- | --- | --- | --- |
| N0 | P0 | 63.86±0.11a | 69.52±0.08ab | 75.17±0.08abcd | 7.58±0.13ab | 0.75±0.12fg | 9.86±0.01f |
|  | P1 | 63.62±0.13ab | 69.24±0.07bcd | 75.65±0.03abc | 7.89±0.58ab | 1.30±0.16ef | 16.85±0.03def |
|  | P2 | 63.95±0.24a | 69.44±0.22abc | 76.23±0.57a | 8.20±0.15ab | 1.73±0.23cde | 21.13±0.03cdef |
|  | P3 | 63.83±0.10ab | 69.20±0.10cd | 75.86±0.31ab | 7.62±0.67ab | 3.12±0.38a | 42.12±0.09ab |
| N1 | P0 | 63.66±0.32ab | 69.45±0.05abc | 75.64±0.35abc | 7.23±0.30ab | 1.66±0.21de | 23.13±0.04cdef |
|  | P1 | 63.40±0.05ab | 69.08±0.07de | 75.92±0.20ab | 8.96±0.41a | 2.23±0.09bcd | 24.85±0.00cde |
|  | P2 | 62.84±0.02ab | 68.70±0.05f | 74.41±0.64cd | 7.50±0.73ab | 2.42±0.15bc | 32.97±0.05abc |
|  | P3 | 63.94±0.04a | 69.27±0.06bcd | 75.49±0.07abc | 7.77±0.04ab | 1.88±0.10cde | 24.16±0.01cdef |
| N2 | P0 | 62.29±1.62b | 69.60±0.07a | 75.73±0.19ab | 7.80±0.06ab | 2.09±0.21cd | 26.78±0.03cd |
|  | P1 | 63.87±0.13a | 68.86±0.02ef | 74.68±0.62bcd | 6.79±1.56b | 2.82±0.18ab | 44.06±0.07a |
|  | P2 | 64.07±0.02a | 69.40±0.07abc | 74.97±0.11abcd | 8.05±0.33ab | 2.04±0.28cd | 25.12±0.02cd |
|  | P3 | 63.68±0.03ab | 68.99±0.02cde | 75.23±0.15abc | 8.17±0.06ab | 2.28±0.16bcd | 27.89±0.02bcd |
| N3 | P0 | 63.67±0.09ab | 68.77±0.09ef | 73.88±0.20d | 4.73±0.05c | 0.49±0.11g | 10.41±0.03ef |
|  | P1 | 63.61±0.12ab | 68.82±0.11ef | 74.63±0.15bcd | 7.13±0.03ab | 2.31±0.14bcd | 32.43±0.02abc |
|  | P2 | 63.66±0.02ab | 68.75±0.06f | 75.33±0.45abc | 8.51±0.70ab | 2.04±0.00cd | 24.22±0.02cdef |
|  | P3 | 63.41±0.02ab | 68.860.01±ef | 75.02±0.49abcd | 7.31±0.52ab | 1.73±0.18cde | 24.11±0.04cdef |
| F (N) | | 1.18ns | 59.28** | 11.60** | 5.07** | 21.01** | 7.99** |
| F (P) | | 0.99ns | 23.00** | 0.95ns | 7.06** | 48.37** | 16.95** |
| F (N×P) | | 3.02* | 14.80** | 6.29** | 5.31** | 19.99** | 10.71** |

Note: Different letters within the same treatment in the figure indicate significant differences at the P=0.05 level;N and P in the F value are nitrogen application rate and Phosphorus application rate. N×P is the interaction between nitrogen application rate and Phosphorus application rate. * and ** represent significant levels of 0.05 and 0.01, respectively.

**Table S3-2.** Response of Jingyou 781 rice DSC content to nitrogen and phosphorus regulation

| Treat | | To (℃) | Tp (℃) | Tc (℃) | ∆Hg (J/g) | ∆Hr (J/g) | R (%) |
| --- | --- | --- | --- | --- | --- | --- | --- |
| N0 | P0 | 64.82±0.01de | 70.65±0.01abc | 77.33±0.12bcde | 8.21±0.12abc | 2.70±0.37abcd | 32.74±0.04bc |
|  | P1 | 65.46±0.09abc | 71.21±0.00ab | 78.54±0.61ab | 7.17±0.03bcd | 1.39±0.00fg | 19.39±0.00def |
|  | P2 | 64.93±0.02cde | 70.51±0.00bc | 76.97±0.16cdef | 7.68±0.43abcd | 3.00±0.04ab | 39.16±0.02ab |
|  | P3 | 65.43±0.16abcd | 71.20±0.29ab | 77.62±0.00abcd | 7.49±0.48abcd | 1.84±0.32def | 24.32±0.03cdef |
| N1 | P0 | 63.14±0.16f | 69.07±0.06d | 75.89±0.19ef | 8.78±0.41ab | 2.21±0.17bcdef | 25.08±0.01cdef |
|  | P1 | 65.40±0.01abcd | 71.00±0.02ab | 78.43±0.33abc | 9.43±0.07a | 2.94±0.05abc | 31.14±0.00bcd |
|  | P2 | 65.35±0.06bcde | 70.83±0.01ab | 77.56±0.05abcd | 8.87±0.35ab | 2.21±0.21bcdef | 24.79±0.01cdef |
|  | P3 | 65.82±0.06ab | 71.41±0.06a | 77.82±0.85abcd | 8.51±1.45abc | 2.02±0.01cdef | 24.72±0.04cdef |
| N2 | P0 | 65.99±0.11a | 71.29±0.07a | 78.04±0.70abcd | 6.61±0.05cd | 3.20±0.71a | 48.22±0.10a |
|  | P1 | 65.48±0.07abc | 70.98±0.07ab | 76.79±0.20def | 8.37±0.27abc | 2.04±0.35bcdef | 24.22±0.03cdef |
|  | P2 | 64.78±0.56e | 69.92±0.74c | 75.58±0.48f | 5.85±0.05d | 0.81±0.02g | 13.87±0.00f |
|  | P3 | 63.11±0.05f | 69.15±0.01d | 75.65±0.09f | 7.91±0.38abc | 2.07±0.07bcdef | 26.14±0.00cde |
| N3 | P0 | 65.44±0.14abc | 70.84±0.12ab | 78.06±0.73abcd | 6.58±0.20cd | 1.57±0.06fg | 23.86±0.00cdef |
|  | P1 | 65.37±0.01bcde | 70.67±0.01abc | 76.76±0.35def | 8.27±0.97abc | 1.66±0.16efg | 20.17±0.00def |
|  | P2 | 65.32±0.18bcde | 70.67±0.16abc | 77.34±0.46bcde | 8.24±0.08abc | 1.30±0.09fg | 15.74±0.01ef |
|  | P3 | 65.79±0.00ab | 71.32±0.01a | 79.11±0.01a | 9.16±0.78a | 2.57±0.33abcde | 27.92±0.01bcd |
| F (N) | | 23.10** | 13.08** | 14.37** | 14.62** | 7.35** | 7.44** |
| F (P) | | 16.55** | 10.75** | 5.17** | 4.39* | 7.25** | 14.43** |
| F (N×P) | | 70.15** | 31.28** | 12.77** | 5.06** | 17.02** | 16.38** |

Note: Different letters within the same treatment in the figure indicate significant differences at the P=0.05 level;N and P in the F value are nitrogen application rate and Phosphorus application rate. N×P is the interaction between nitrogen application rate and Phosphorus application rate. * and ** represent significant levels of 0.05 and 0.01, respectively.

**Table S4.** Effects of nitrogen and phosphorus regulation on starch crystallinity

| Treat | | Deyou 4727 | Jingyou 781 |
| --- | --- | --- | --- |
| N0 | P0 | 24.60±0.56bc | 24.48±0.81bc |
|  | P1 | 21.25±0.71d | 20.58±0.69bc |
|  | P2 | 26.98±0.62ab | 22.43±0.55c |
|  | P3 | 27.35±0.88ab | 22.95±0.84c |
| N1 | P0 | 25.67±0.84ab | 25.78±0.61b |
|  | P1 | 29.20±0.77a | 19.48±0.57c |
|  | P2 | 26.44±0.83ab | 27.48±0.98b |
|  | P3 | 24.61±0.87c | 28.06±0.58b |
| N2 | P0 | 23.29±0.35bc | 22.42±0.25d |
|  | P1 | 27.01±0.67ab | 29.80±1.15a |
|  | P2 | 24.12±0.84bc | 31.22±1.01a |
|  | P3 | 23.02±0.58c | 32.81±1.04a |
| N3 | P0 | 20.66±0.64c | 26.45±0.71ab |
|  | P1 | 23.95±0.81bc | 23.14±1.04b |
|  | P2 | 23.19±0.61c | 31.19±0.88a |
|  | P3 | 28.80±0.49ab | 35.57±1.48a |

Note: N, nitrogen application rate; P. Phosphorus application rate; The same column of different lowercase letters indicates that there is a significant difference at the 5% level between the same panicle position and different varieties.

**Table S5-1.** Response of starch grain morphology to nitrogen and phosphorus regulation in Deyou 4727 rice

| Treat | | grain size | | | grain type | |
| --- | --- | --- | --- | --- | --- | --- |
|  |  | small-sized starch granules | medium-sized starch granules | large-sized starch granules | circular | rule-based |
| N0 | P0 | 18.95±1.36ef | 46.24±1.79efg | 34.80±0.79cd | 14.87±0.46a | 85.13±0.46i |
|  | P1 | 12.73±0.45i | 45.68±1.46efg | 41.59±1.04b | 10.68±0.95de | 89.32±0.95def |
|  | P2 | 14.86±0.60h | 41.71±0.57h | 43.43±0.25a | 12.29±0.67bc | 87.71±0.67efgh |
|  | P3 | 15.89±0.53h | 47.92±0.94cde | 36.19±0.44c | 10.51±0.57de | 89.98±0.72bcde |
| N1 | P0 | 19.74±0.77ef | 38.21±0.91i | 42.05±0.46ab | 12.82±0.75abc | 87.18±0.75fghi |
|  | P1 | 20.60±0.26de | 48.84±0.62cd | 30.56±0.74e | 8.64±1.01f | 91.36±1.01abc |
|  | P2 | 17.76±0.87fg | 54.93±0.55b | 27.30±1.11fg | 9.21±0.28ef | 90.79±0.28bcd |
|  | P3 | 16.52±0.56gh | 50.45±0.65c | 33.03±0.59d | 10.51±0.62de | 89.49±0.62cdef |
| N2 | P0 | 26.80±0.56b | 45.82±1.64fg | 27.38±2.18f | 11.24±1.93cde | 88.76±1.93def |
|  | P1 | 23.37±0.36c | 55.17±0.58b | 21.46±0.45h | 8.05±0.28f | 91.95±0.28ab |
|  | P2 | 20.69±0.76de | 53.32±0.93b | 25.99±0.82g | 10.88±0.63de | 89.12±0.63def |
|  | P3 | 19.38±0.63ef | 61.88±0.53a | 18.75±0.25i | 7.19±0.41f | 93.13±0.91a |
| N3 | P0 | 22.35±1.62cd | 44.24±1.49g | 33.41±0.14cd | 11.52±0.49cde | 88.71±0.81defg |
|  | P1 | 21.90±0.16cd | 43.81±0.32g | 34.29±0.48cd | 7.94±0.58f | 92.06±0.58ab |
|  | P2 | 18.86±0.82ef | 55.89±0.69b | 25.25±0.39g | 14.48±0.71a | 85.52±0.71hi |
|  | P3 | 37.91±0.27a | 45.49±0.22efg | 16.6±0.081i | 13.36±0.38ab | 86.64±0.78ghi |
| F (N) | | 255.22** | 112.38** | 574.74** | 23.57** | 19.50** |
| F (P) | | 56.80** | 108.16** | 146.64** | 36.91** | 33.13** |
| F (N×P) | | 90.81** | 54.93** | 93.76** | 9.99** | 10.24** |

Note: Different letters within the same treatment in the figure indicate significant differences at the P=0.05 level;N and P in the F value are nitrogen application rate and Phosphorus application rate. N×P is the interaction between nitrogen application rate and Phosphorus application rate. * and ** represent significant levels of 0.05 and 0.01, respectively.

**Table S5-2.** Response of starch grain morphology to nitrogen and phosphorus regulation in Jingyou 781 rice

| Treat | | grain size | | | grain type | |
| --- | --- | --- | --- | --- | --- | --- |
|  |  | small-sized starch granules | medium-sized starch granules | large-sized starch granules | circular | rule-based |
| N0 | P0 | 11.63±0.41g | 47.74±0.16b | 40.64±0.57b | 13.13±0.69ab | 86.87±0.69de |
|  | P1 | 23.40±1.03c | 36.29±1.12ef | 40.31±0.12b | 14.10±0.26a | 85.90±0.26e |
|  | P2 | 29.10±1.01a | 37.29±0.48ef | 33.61±1.00def | 12.40±0.43ab | 87.60±0.43de |
|  | P3 | 24.05±0.16c | 31.17±1.62h | 44.79±1.55a | 13.46±0.82ab | 86.54±0.82de |
| N1 | P0 | 24.36±0.62c | 33.81±0.46g | 41.84±0.64b | 9.63±0.46cd | 90.37±0.46bc |
|  | P1 | 27.11±1.17b | 38.37±0.27de | 34.53±0.94d | 13.09±0.30ab | 86.91±0.30de |
|  | P2 | 22.63±0.58c | 43.03±0.25c | 34.34±0.39de | 11.35±0.76bc | 88.65±0.76cd |
|  | P3 | 21.81±c0.87d | 35.53±0.76fg | 42.65±0.63b | 12.36±0.86ab | 87.64±0.86de |
| N2 | P0 | 19.70±1.15de | 48.22±0.53b | 32.08±1.29efg | 7.58±0.93de | 92.42±0.93ab |
|  | P1 | 19.66±1.33de | 43.41±1.37c | 36.93±1.20c | 11.93±1.29ab | 88.07±1.29de |
|  | P2 | 24.12±0.50c | 44.61±0.59c | 31.27±0.44fgh | 8.65±1.18d | 91.35±1.18b |
|  | P3 | 18.72±0.82e | 39.92±1.48d | 41.36±0.66b | 9.22±0.46d | 90.78±0.46b |
| N3 | P0 | 14.79±0.96f | 53.40±0.83a | 31.81±1.03efg | 9.87±0.37cd | 90.13±0.37b |
|  | P1 | 23.18±0.63c | 47.70±0.79b | 29.12±0.17g | 9.12±1.02d | 90.88±1.02b |
|  | P2 | 15.90±0.47f | 54.05±1.76a | 30.05±1.36fg | 8.27±0.58de | 91.73±0.58ab |
|  | P3 | 19.73±0.78de | 48.40±0.57b | 31.86±0.41efg | 6.48±0.39e | 93.52±0.39a |
| F (N) | | 62.77** | 337.63** | 166.56** | 69.75** | 69.75** |
| F (P) | | 76.90** | 90.64** | 109.37** | 12.87** | 12.87** |
| F (N×P) | | 50.31** | 33.77** | 19.89** | 5.78** | 5.78** |

Note: Different letters within the same treatment in the figure indicate significant differences at the P=0.05 level;N and P in the F value are nitrogen application rate and Phosphorus application rate. N×P is the interaction between nitrogen application rate and Phosphorus application rate. * and ** represent significant levels of 0.05 and 0.01, respectively.

**Table S6.** Correlation of nitrogen and phosphorus application with appearance quality and starch granule characteristics (Deyou 4727)

| Index | N | P | CD | CR |
| --- | --- | --- | --- | --- |
| Diffraction angle 2θ | 0.05 | 0.28 | 0.17 | 0.2 |
| Small starch granules | 0.65** | 0.06 | -0.40** | -0.01 |
| Middle starch granules | 0.21 | 0.44** | -0.25 | -0.01 |
| Large starch granules | -0.63** | -0.38** | 0.48** | 0.02 |
| Round starch grains | -0.09 | -0.17 | 0.40** | 0.40** |
| Regular starch granules | 0.07 | 0.21 | -0.35* | -0.35* |

Note: N, nitrogen application rate; P. Phosphorus application rate; CD, chalkiness Degree; CR, chalky grain rate; *, P ≤ 0.05, * *, P ≤ 0.01.

**Table S7.** Correlation of nitrogen and phosphorus application with appearance quality and starch granule characteristics (Jingyou 781)

| Index | N | P | CD | CR |
| --- | --- | --- | --- | --- |
| Diffraction angle 2θ | 0.60** | 0.28 | -0.47** | -0.54** |
| Small starch granules | -0.36* | 0.18 | 0.47** | 0.17 |
| Middle starch granules | 0.74** | -0.32* | -0.71** | -0.30* |
| Large starch granules | -0.68** | 0.27 | 0.54** | 0.26 |
| Round starch grains | -0.79** | -0.05 | 0.69** | 0.51** |
| Regular starch granules | 0.79** | 0.05 | -0.69** | -0.51** |

Note: N, nitrogen application rate; P. Phosphorus application rate; CD, chalkiness Degree; CR, chalky grain rate; *, P ≤ 0.05, * *, P ≤ 0.01.
